# Overcoming the exciton binding energy in two-dimensional perovskite nanoplatelets by attachment of conjugated organic chromophores

**DOI:** 10.1038/s41467-020-15869-7

**Published:** 2020-04-20

**Authors:** María C. Gélvez-Rueda, Magnus B. Fridriksson, Rajeev K. Dubey, Wolter F. Jager, Ward van der Stam, Ferdinand C. Grozema

**Affiliations:** 1 Department of Chemical Engineering, Delft University of Technology, Van der Maasweg 9, 2629 HZ Delft, The Netherlands; 2 Present Address: POLYMAT, Basque Center for Macromolecular Design and Engineering, University of the Basque Country UPV/EHU, Avenida de Tolosa 72, 20018 Donostia-San Sebastian, Spain; 3 Present Address: Inorganic Chemistry and Catalysis, Debye Institute for Nanomaterials Science, Utrecht University, Universiteitsweg 99, 3584 CG Utrecht, The Netherlands

**Keywords:** Electronic materials, Electron transfer, Two-dimensional materials

## Abstract

In this work we demonstrate a novel approach to achieve efficient charge separation in dimensionally and dielectrically confined two-dimensional perovskite materials. Two-dimensional perovskites generally exhibit large exciton binding energies that limit their application in optoelectronic devices that require charge separation such as solar cells, photo-detectors and in photo-catalysis. Here, we show that by incorporating a strongly electron accepting moiety, perylene diimide organic chromophores, on the surface of the two-dimensional perovskite nanoplatelets it is possible to achieve efficient formation of mobile free charge carriers. These free charge carriers are generated with ten times higher yield and lifetimes of tens of microseconds, which is two orders of magnitude longer than without the peryline diimide acceptor. This opens a novel synergistic approach, where the inorganic perovskite layers are combined with functional organic chromophores in the same material to tune the properties for specific applications.

## Abstract (web summary)

Functionalizing two-dimensional (2D) hybrid perovskites with organic chromophores is a novel approach to tune their optoelectronic properties. Here, the authors report efficient charge separation and conduction in 2D hybrid perovskite nanoplatelets by incorporating an electron acceptor chromophore.

## Introduction

Two-dimensional (2D) hybrid organic–inorganic perovskites are an emerging class of materials with potential application in a broad range of opto-electronic devices, such as solar cells, light-emitting diodes, photo-detectors, spintronics, waveguides, nano-lasers and photo-catalysis^1–3^. 2D hybrid perovskites differ from three-dimensional perovskites, which are currently among the most promising materials for solar cell applications, due to the presence of large organic cations that lead to the formation of 2D layers of inorganic metal-halide octahedra separated by the organic cations. The large organic cations improve the stability under ambient conditions compared to the 3D analogues, and offer novel possibilities to tune the opto-electronic properties^1^. Until now, the properties of 2D hybrid perovskites have been tuned by changing the nature of the large organic cations (distorting the inorganic metal-halide octahedrals)^4–10^ or by combining them with small organic cations to adjust the number of stacked inorganic octahedral layers (changing the dielectric environment)^9,11–13^. In the majority of previous studies, the large organic cation is an alkylammonium (mostly butyl) or phenyl-alkylammonium^14^, which lack specific functionality and do not directly contribute to the opto-electronic properties other than by affecting the structure of the inorganic layers.

There are, however, endless possibilities to engineer the electronic structure of 2D hybrid perovskites by introduction of functional organic molecules, for instance strong electron donors or acceptors. As we have shown in a recent theoretical study^15^, such donor or acceptor molecules can directly contribute to the electronic bands. This could result in enhanced charge separation, compared to current 2D perovskites where the organic cation merely acts as a non-functional dielectric spacer-layer, leading to a high exciton binding energy (ranging from ~190–400 meV for pure 2D, down to ~80 meV for quasi-2D system with four inorganic layers between the organic cations^16–19^), and hence inefficient optical generation of charges.

In some recent studies, functional organic cations such as conjugated molecules^20,21^ and charge-transfer complexes^22,23^ have been introduced in 2D hybrid perovskites. However, only limited effects on the opto-electronic properties have been reported^20,22^. Some promising behavior has been observed, such as an increased out-of-plane conductivity by tunneling through the organic cations^21^ and photoluminescence (PL) quenching (without clarifying whether it is caused by energy or charge transfer)^20^. It should be noted that it is hard to predict a priori whether a stable 2D materials will be formed, and other dimensionalities are sometimes obtained because of the interactions between the organic chromophores^24,25^.

An attractive approach to explore the effect of organic molecules on the photophysics of 2D perovskites is the use of colloidal perovskite platelets^26–28^. This reduces the packing requirements of the organic molecules as only a small fraction of the ligands can be replaced with the conjugated molecules. Introduction of conjugated molecules that are strong electron donors or acceptors can be an approach to the formation of long-lived mobile charge carriers. While charge separation may be achieved by adding donor and acceptor molecules in solution^29^, the eventual application in the solid state requires that they contain binding groups with affinity for the nanoplatelets (NPLs)^30^. For example, perylene diimides (PDI) are well-known electron acceptors used in organic electronics and photovoltaics^31–33^. Their high electron affinity and efficient charge and excited state transport, combined with their exceptional thermal and photochemical stability, makes them ideal candidates to achieve charge separation in 2D hybrid perovskites and potentially develop solution-based and solid-state opto-electronic devices.

In this work, we explore the introduction of functional organic cations in 2D perovskites in order to enhance their functionality by, for example, inducing change separation from the inorganic octahedral layer into the organic chromophores. Specifically, we have replaced the non-functional organic ligands at the surface of colloidal NPLs by strong electron-accepting PDIs^34^. The NPLs are colloidal quasi-2D cesium lead bromide NPLs consisting of four layers of lead bromide octahedra (CsPbBr_3_ NPLs, *n* = 4)^26–28^. In contrast to previous studies^29^, the acceptors used in this work are not merely added in solution but have been modified to contain an ammonium group so that it can coordinatively attach to the surface of the platelets^30^. Attachment to the same material overcomes morphological issues that can arise when heterojunctions of organic–inorganic materials are made^35^. Using a combination of ultra-fast spectroscopy and time-resolved conductivity techniques, we unequivocally show that the introduction of electron-accepting PDIs leads to strongly enhanced charge separation and the formation of long-lived charge carriers useful for opto-electronic devices. This opens up a novel synergistic route to materials that are tuned for specific applications by combining the inorganic perovskite layers with functional organic chromophores. This concept is not limited to inducing charge separation but can also be extended to the use of chiral molecules for circularly polarized light detection^36^ or singlet fission/up-conversion chromophores to enhance light absorption^37,38^.

## Results

### Synthesis and characterization

**Fig. 1:**
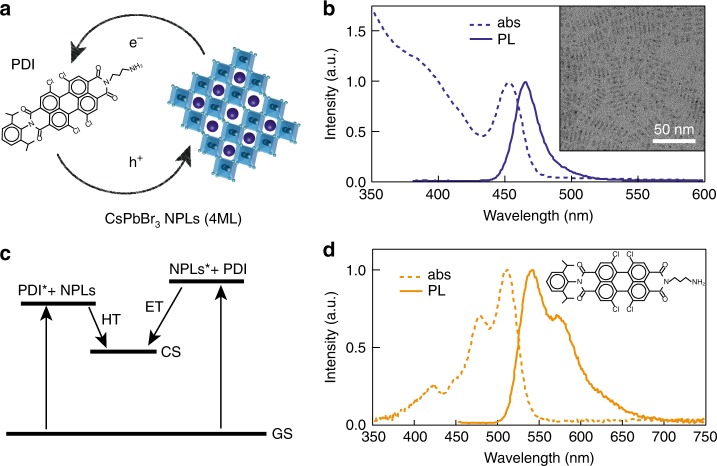
CsPbBr_3_ nanoplatelet–perylene diimide hybrids. **a** Schematic representation of CsPbBr_3_ NPLs and PDI molecules charge transfer. **b** Optical absorption (dashed line) and photoluminescence (PL) emission (full line) spectra of CsPbBr_3_ NPLs (4 monolayers (ML)). Transmission electron microscopy image of the NPLs (inset). **c** Schematic representation of the charge-transfer processes indicating the formation of the charge separated state (CS) by hole transfer (HT) from the PDI excited state (PDI*) to NPLs and electron transfer (ET) from the NPLs excited state (NPLs*) to PDI. **d** Optical absorption (dashed line) and PL emission (full line) spectra of PDI molecules.

The perovskite NPLs and PDI molecules were synthesized with the aim to selectively photo-excite both components at different wavelengths and study the possibility of electron transfer (ET) from the NPLs to the PDI molecules, as well as hole transfer (HT) from the PDI molecules to the NPLs (Fig. 1a, c). 2D colloidal CsPbBr_3_ NPLs (5 × 10 nm, thickness ~1.5 nm, 4 atomic monolayers (4ML)) were synthesized through a recrystallization method in which Cs-oleate and PbBr_2_ crystallize when acetone is added as antisolvent^26^. Their optical absorption and PL are shown in Fig. 1b. By careful synthetic control, monodisperse atomically smooth NPLs are obtained (see TEM in Fig. 1b, inset) with a pronounced thickness-dependence of the excitonic absorption and fluorescence (Supplementary Fig. 1 and Supplementary Note 1). 4ML CsPbBr_3_ NPLs were chosen here as their main exciton peak (450 nm) does not overlap with the absorption of the PDI (520 nm, Fig. 1d and Supplementary Fig. 2)^31^. The colloidal NPLs are dispersed in hexane due to their oleate organic capping layer. Subsequently, these ligands were partially replaced by a strongly electron-accepting PDI derivative (Fig. 1d) to achieve efficient charge separation. The PDI with the alkylammonium linker at the imide position and four chlorine atoms in the “bay-area” was synthesized in two steps from a perylene monoimide monoanhydride derivative as described in the methods section and Supplementary Figs. 3, 4 and 5^34^. The alkylammonium group is introduced so that it can coordinatively attach to the surface of the NPLs^30^, while the chlorine atoms ensure sufficient solubility in common organic solvents such as dichloromethane (DCM). The mixed CsPbBr_3_ NPLs + PDI solution is prepared by adding the PDI stock solution in DCM to the dispersed CsPbBr_3_ NPLs solution in hexane under continuing stirring and mild heat (50 °C). We have estimated from optical density measurements in hexane^39^ that ~90 PDI molecules coordinate to the surface of the CsPbBr_3_ NPLs (Supplementary Note 2). This value is reasonable as is highly likely that on the dynamic NPLs surface^30^ there are also oleic acid anionic ligands on Cs^+^ states, while the PDI molecules would act as cationic ligands on PbBr states. We are convinced that the most direct proof of the attachment of the PDIs to the perovskite NPLs is shown by our TA measurements photoexciting the PDI (510 nm excitation) as described below in this work. Attempts to show the attachment by nuclear magnetic resonance (NMR) measurements are far from conclusive in this case (and in many cases in literature^40,41^) because there is always a certain fraction of the PDIs (or ligands, such as oleic acid) in solution. Moreover, the binding-equilibrium conditions in the type of solvent (deuterated chloroform) required for NMR measurements are different than in hexane. Full details on the synthesis and characterization of the 2D NPLs and PDI can be found in the Methods section.

### Picosecond photoluminescence measurements

**Fig. 2:**
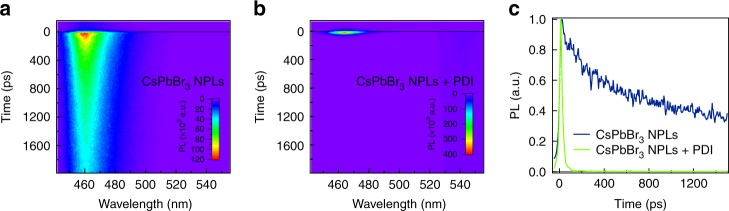
Picosecond photoluminescence measurements on photoexcitation at 400 nm. **a** Photoluminescence (PL) of CsPbBr_3_ NPLs as a function of time and wavelength. **b** PL of CsPbBr_3_ NPLs + PDI hybrid as a function of time and wavelength. **c** Comparison of the temporal decay of the fluorescence of CsPbBr_3_ NPLs (4ML) and CsPbBr_3_ NPLs + PDI hybrid at 460 nm. The fluorescence quenching in CsPbBr_3_ NPLs + PDI hybrid may be a result of electron transfer to PDI.

To study the effect of the attachment of PDI molecules to the CsPbBr_3_ NPLs on the lifetime of excitons, we have performed picosecond PL measurements using a streak camera. The solutions are photoexcited at a wavelength of 400 nm, reaching a high absorption in the CsPbBr_3_ NPLs while minimizing the direct absorption in the PDI. In Fig. 2a, the PL spectrum for the NPLs without attached PDI is shown as a function of time. The PL decay in the NPLs lives beyond 1800 ps but the decay cannot be described by a single exponent. Because of the limited maximum time window of our experimental setup, we are not able characterize both decay components accurately and therefore only report the first half-life time (~600 ps), which is comparable to values in literature^29^. Addition of the PDI molecules leads to striking changes. The shape of the fluorescence spectrum remains the same but the lifetime is strongly reduced to ~16 ps (Fig. 2b, c, Supplementary Fig. 6). This suggests that the excitons in the NPLs decay rapidly by electron transfer to the electron-accepting PDIs. In addition, we observe a weak emission from the PDI (540 nm) with a lifetime that is the same as for PDIs in solution (Supplementary Figs. 7 and 8). As there is no in-growth of this feature on the time scale of the decay of the fluorescence from the platelets, we attribute this to fluorescence from free PDI molecules in the solution, rather than energy transfer from the NPLs. This is confirmed by transient absorption (TA) measurements as described below.

### Electron transfer: transient absorption on photoexcitation at 400 nm

**Fig. 3:**
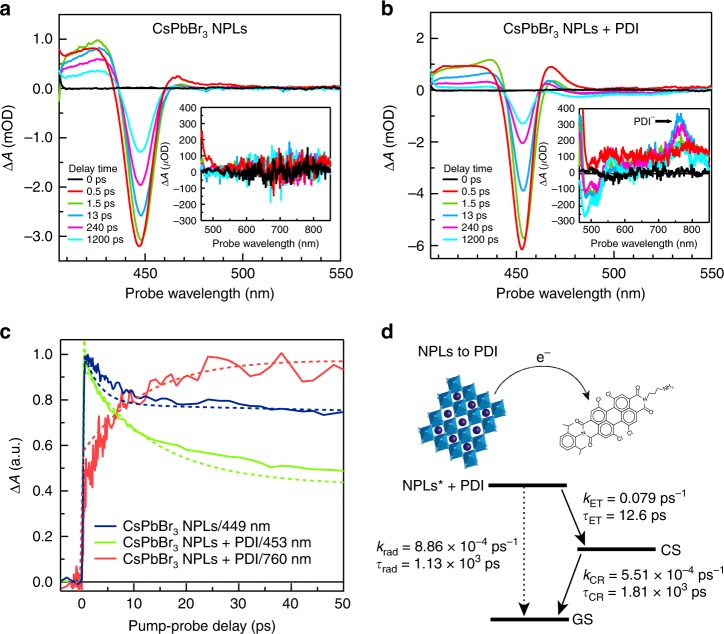
Electron transfer from CsPbBr_3_ nanoplatelets to perylene diimide chromophores upon exciting at 400 nm. **a** Transient absorption (TA) spectrum of CsPbBr_3_ NPLs. The inset shows the 465–850 nm spectral range. **b** TA spectrum of CsPbBr_3_ NPLs + PDI hybrid. The inset shows the 465–850 nm spectral range, showing the spectral features of the PDI anion (PDI^−^). **c** Comparison of temporal dynamics of main exciton bleach of CsPbBr_3_ NPLs (~449 nm), the main exciton bleach of CsPbBr_3_ NPLs + PDI hybrid exciton bleach (453 nm) and the PDI anion (PDI^−^) photoinduced absorption growth (~760 nm). Solid lines represent the experimental data while dotted lines are the result of the target global analysis. **d** Kinetic model used for the target global analysis for electron transfer (ET) from NPLs to the PDI molecules in CsPbBr_3_ NPLs + PDI hybrid (for details, see Supplementary Note 3).

To determine whether the deactivation of the excitons in the NPLs is indeed due to electron transfer from the CsPbBr_3_ NPLs to PDI, we have studied the exciton dynamics by femtosecond TA measurements. The solutions were photoexcited at a wavelength of 400 nm with a ~180 fs laser pulse, similar to the fluorescence measurements described above. Subsequently, the changes in the optical absorption spectrum due to photoexcitation were monitored using short, broadband pulses that are obtained from continuum generation in a CaF_2_ crystal (340–900 nm). All TA measurements were performed at room temperature with low absorbed pump fluences (~3.5 × 10^12^ photons/(cm^2^ pulse)) in order to avoid second order effects due to generation of multiple excitons in a single platelet. The TA spectra of CsPbBr_3_ NPLs and CsPbBr_3_ NPLs + PDI are shown in Fig. 3 at different times after the excitation pulse.

As shown in Fig. 3a, the TA spectrum of CsPbBr_3_ NPLs exhibit the typical features found in literature for these materials^29^: a negative exciton bleach (XB) at ~448 nm accounting for band-edge filling by photogenerated excitons superimposed on a broad photoinduced absorption (PA) between 380 and 480 nm. The shape of the TA spectrum does not change with the delay time, confirming the presence of only long-lived single-exciton states (Supplementary Fig. 9)^29^. The kinetics of the ground state bleach at 448 nm are shown in Fig. 3c. We analyzed the TA spectrum with global and target analysis using the open source software Glotaran^42^. In this procedure, the TA spectra are parametrized in time as a linear combination of absorption spectra from the different transient species that interconvert into each other according to a pre-defined kinetic scheme (for details, see Supplementary Note 3)^43^. The target analysis was applied to the 400 nm excitation data with the kinetic scheme shown in Supplementary Fig. 14. The signal grows within time resolution of the TA setup and decays bi-exponentially with decay time constants of 3 and 1470 ps. The fast decay component corresponds to fast trapping due to surface defects on perovskite NPLs^30^. The second time component is comparable to the exciton lifetime obtained from the PL measurements discussed above.

Introduction of the PDI molecules in the ligand shell of the NPLs again leads to striking changes in the TA spectrum (Fig. 3b). The XB feature at 453 nm has the same shape as observed for the CsPbBr_3_ NPLs (Fig. 3a), however, it decays much faster. This is also shown in Fig. 3c, where the kinetics at the maximum of the exciton bleach of the NPLs with and without PDI are compared. In addition, extra features appear in the TA spectrum that are not present in the pure CsPbBr_3_ NPLs (Fig. 3a, b, insets). The first is a reduced absorption (bleach) in the region from 470 to 540 nm, corresponding to the ground state absorption of the PDI molecule (Fig. 1d). This indicates a decrease of the population of PDI molecules in their ground state, due to electron transfer (ET) from the CsPbBr_3_ NPLs to the PDI, resulting in a charge separated (CS) state where the interaction between the electron in the PDI molecules and the holes in the NPLs is virtually zero as the holes are fully delocalized in the NPLs. The second feature is a photoinduced absorption (PA) with a maximum at 760 nm. The shape and position of this second feature are close to the known absorption spectrum of the PDI anion (PDI^−^)^44^, although it is shifted to longer wavelengths because of the twisting of the PDI^−^ core caused by the introduction of chlorines in the bay area. Note, that the photoinduced absorption of the PDI^−^ in Fig. 3b inset is different from the induced absorption due to the excited state of free PDI molecules in solution (PDI*) centered at ~800 nm (Supplementary Fig. 10)^44^. In Fig. 3c, the kinetics from the PA of the PDI^−^ at 760 nm are also shown. The occurrence of electron transfer also explain the fast initial decay of the bleach of the NPLs mentioned above. It is well-documented that excitons in NPLs and nanocrystals exhibit a larger ground state bleach than charged particles, but at the same wavelength ^45^.

The TA data of CsPbBr_3_ NPLs + PDI was analyzed by global target analysis based on the kinetic scheme in Fig. 3d. This analysis yields the rates of the different processes that occur after photoexcitation, summarized in Fig. 3d. The fits are compared to the experimental data in Fig. 3c. From this analysis, we determined that electron transfer (ET) from the NPLs to the PDIs proceeds with a time constant, *τ*_ET_ = 12.6 ps, while decay of this CS state back to the ground state (GS) by charge recombination (CR) occurs with a time constant of *τ*_CR_ = 1800 ps. The value of τ_ET_ is in good agreement with the exciton lifetime obtained from the fluorescence measurements (16 ps). All these features unequivocally show that the excitons in the CsPbBr_3_ NPLs decay by ET to the PDI molecules. The efficiency of electron transfer can be estimated to be ~52% comparing the absorbance of the PDI molecules in the excited state ([PDI*]) at ~520 nm to the absorbed photons of CsPbBr_3_ NPLS + PDI at 400 nm (for details, see Supplementary Note 4).

### Hole transfer: transient absorption on photoexcitation at 510 nm

In order to determine whether the inverse process, HT from the PDI molecules to the CsPbBr_3_ NPLs also takes place, we have studied the exciton dynamics by TA measurements photoexciting at 510 nm. This wavelength corresponds to the maximum in absorption of the PDI, while there is no absorption by the NPLs. As before, the absorbed pump fluences were kept sufficiently low (~2.7 × 10^12^ photons/(cm^2^ pulse)) to avoid multiexciton generation. In addition, for reference, we measured the TA spectra of free CsPbBr_3_ NPLs and free PDI molecules (Supplementary Figs. 11 and 12). For free CsPbBr_3_ NPLs, no changes in the absorption are observed, as expected, since the NPLs do not absorb at 510 nm. For isolated PDI molecules, the TA spectrum exhibits the typical features of the PDI excited state (PDI*): a bleach of the ground state absorption at wavelengths between 470 and 540 nm, stimulated emission at ~580 nm and a broad photoinduced absorption (PA) from 690 to 900 nm with a narrow maximum at 800 nm^31,44,46^. PDI* decays with a time constant of 4 ns, which is close to the fluorescence lifetime determined for this compound of *τ*_rad_ = 3.8 ns (Supplementary Fig. 13).

**Fig. 4:**
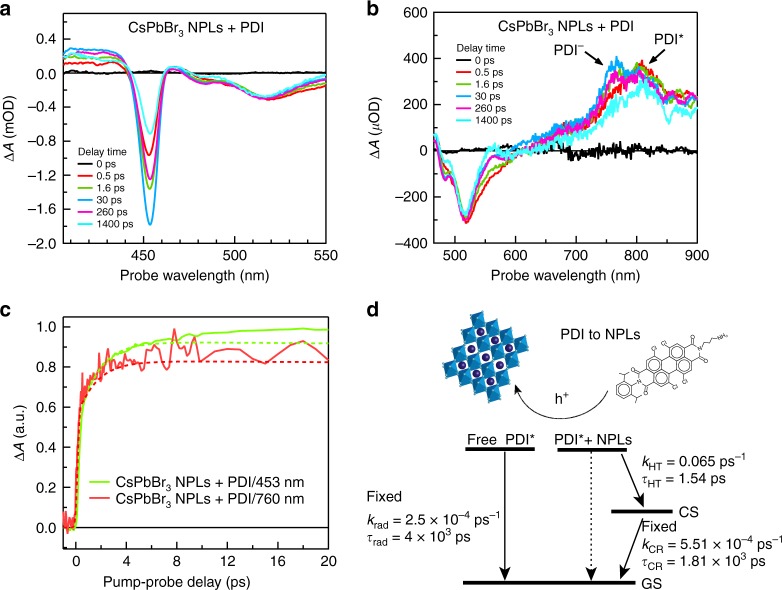
HT from perylene diimide chromophores to CsPbBr_3_ nanoplatelets upon exciting at 510 nm. **a** Transient absorption (TA) spectrum of CsPbBr_3_ NPLs + PDI hybrid between 400 and 550 nm, showing the growth (up to 30 ps) and subsequent decay of the exciton bleach of the NPLs. **b** TA spectrum of CsPbBr_3_ NPLs + PDI hybrid between 465 and 900 nm, showing the spectral features of PDI excited state (PDI*) and PDI anion (PDI^−^). **c** Comparison of the temporal dynamics of the exciton bleach of CsPbBr_3_ NPLs + PDI hybrid exciton bleach (453 nm) and the PDI^−^ photoinduced absorption growth (~762 nm). Solid lines represent the experimental data while dotted lines are the result of the target global analysis. **d** Kinetic model used for the target global analysis for hole transfer (HT) from PDI chromophores to NPLs in CsPbBr_3_ NPLs + PDI hybrid. The ratio of attached PDI vs. PDI in solution was fitted to be 4:6 (for details, see Supplementary Note 3).

In Fig. 4a, b, the TA spectrum of CsPbBr_3_ NPLs + PDI upon excitation at 510 nm is shown. The TA spectra clearly illustrate the hole transfer (HT) from the PDI molecules to the CsPbBr_3_ NPLs. In Fig. 4b, we first observe the instantaneous formation of PDI* by the presence of the PA feature at 800 nm. Subsequently, this initial PDI* evolves (*t* > 1 ps) into the spectrum of the PDI anion (PDI^−^), characterized by photoinduced absorption at ~760 nm. Simultaneously, Fig. 4a shows how the XB from the NPLs at 453 nm grows in time over ~30 ps as holes are transferred from the PDI* to the NPLs. The increase of the XB of the NPLs correlates with the growth of the PDI^−^ as shown in the experimental temporal dynamics, solid lines, as shown in Fig. 3c. In addition, the long lifetime of the XB signal, extending into the nanosecond regime indicates the formation of long-lived charges in the NPLs. These TA spectrum features show the clear presence of two distinct populations of PDI, those attached (decaying very fast by HT) and those in solution (behaving as regular PDIs in solution).

A global and target analysis of the photophysical processes upon excitation at 510 nm was performed using the kinetic scheme shown in Fig. 4d. This scheme accounts for both the PDIs attached to the NPLs (and subsequent HT to the NPLs and formation of the PDI anion) and the unattached PDI molecules in solution. In the analysis, we fixed the decay of free PDIs in solution (*τ*_rad_ ~4 ns, see above) and the decay of the CS state back to the ground state obtained from 400 nm excitation experiments (*τ*_CR_ = 1810 ps). These assumptions lead to a reasonable fit of the kinetics shown by dotted lines in Fig. 4c. The resulting time rate of HT from the PDIs to the NPLs is extremely fast (*τ*_HT_ = 1.5 ps), i.e. one order of magnitude faster than electron transfer (*τ*_ET_ = 12.6 ps) from the NPLs to the PDI molecules as discussed above. The latter is remarkable as the driving force for HT (Δ*G*_CS_) is smaller than for electron transfer as the energy of the initial state (PDI*) is lower, while the final charge separated (CS) state is exactly the same. This suggests that electron transfer takes place in the Marcus inverted region^47^ where a smaller charge-transfer rate is obtained with a larger Δ*G*_CS_ (Fig. 1c). Based on the excited state energies from the optical absorption (Fig. 1b, d), we can assure that the driving force for electron transfer is at least 350 meV. However, the exact driving forces for the system are difficult to estimate due to the unknown stabilization of the oxidation and reduction potentials in the solvent considering the localization of electrons in the PDI molecules and delocalization of holes in the NPLs (for details, see Supplementary Note 5). The efficiency of HT is estimated to be ~54%, which is similar to that of ET. This is surprising considering the faster HT rate determined by the global analysis. Nevertheless, the estimated HT efficiency has a higher error margin due to partial excitation of free PDI molecules.

### Photoconductivity: time-resolved microwave measurements

**Fig. 5:**
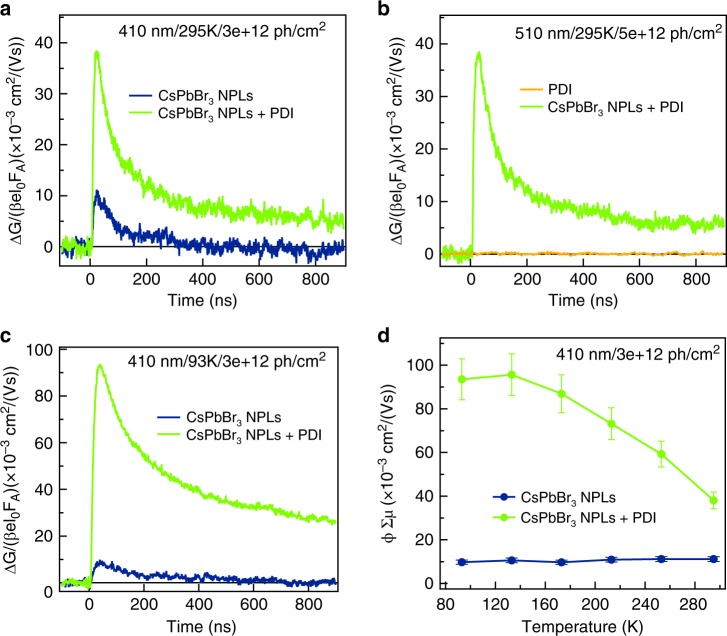
Photoinduced conductivity measurements. **a** Photoinduced conductivity as a function of time at 295 K of CsPbBr_3_ NPLs and CsPbBr_3_ NPLs + PDI hybrid on photoexcitation at 410 nm. **b** Photoinduced conductivity as a function of time at 295 K of PDI and CsPbBr_3_ NPLs + PDI hybrid on photoexcitation at 510 nm. **c** Photoinduced conductivity as a function of time at 93 K of CsPbBr_3_ NPLs and CsPbBr_3_ NPLs + PDI hybrid on photoexcitation at 410 nm. **d** Maximum photoconductivity as a function of temperature of CsPbBr_3_ NPLs and CsPbBr_3_ NPLs + PDI hybrid on photoexcitation at 410 nm. The solutions were drop-casted on quartz substrates. The photon fluence is ~3–5 × 10^12^ photons/cm^2^.

The TA measurements presented above clearly show that charge carriers with lifetimes extending into the nanosecond regime are formed, either on exciting the NPLs or the attached PDI molecules. To establish whether the positive charges in the NPLs are actually mobile on timescales relevant to device application, we have performed photoconductivity measurements using the time-resolved microwave conductivity (TRMC) technique on drop-casted films of the CsPbBr_3_ NPLs and the CsPbBr_3_ NPLs + PDI. In these measurements^48^, microwaves with a frequency close to 10 GHz are used to probe the change in conductivity upon photoexcitation with a ~2.5 ns laser pulse. TRMC measurements are only sensitive to the presence of free mobile charge carriers, trapped charges or neutral Coulomb-bound electron–hole pairs (excitons) will not be detected. It should be noted that the TRCM experiment has a much higher sensitivity (due to the microwave cavity) than the optical TA experiment. This means that we can observe very small fractions of separated charges on longer timescales, while these may not be observed in the TA experiment. Importantly, the photoconductivity is a product of the mobility and dissociation yield of excitons. This yield is determined by the exciton binding energy of the material. Owing to the large exciton binding energy of 2D CsPbBr_3_ NPLs (~260 meV)^29^, a low and short-lived photoconductivity signals is expected for these materials^19^. The photoinduced conductivity is shown as a function of time in Fig. 5a and b upon photoexcitation at 410 nm (exciting the NPLs) and 510 nm (exciting the PDIs), respectively. For CsPbBr_3_, NPLs + PDI high and long-lived photoconductivity signals are observed, both for excitation of the NPLs (410 nm) and PDI (510 nm). This directly shows that the charge separation process shown by the TA measurements above leads to mobile charge carriers in the NPLs. As evident from the similar amplitude and decays kinetics in Fig. 5a and b, the yield and mobility of the charges formed by either hole or electron transfer is similar (as determined above by TA measurements). For comparison, the TRMC transients for the isolated NPLs and PDIs are also shown in Fig. 5a and b, respectively. Photoexcitation of the NPLs without PDI leads to a much lower, short-lived conductivity signal, as expected. Photoexcitation of the pure PDI films yields no measurable conductivity.

In a previous study^19^, we determined that the photoconductivity of 2D hybrid perovskites decreases at low temperature due to the decrease of the yield of dissociation of excitons by thermal energy. If the charge carriers are efficiently separated by electron transfer to the PDI molecules (exciting at 410 nm), the photoconductivity should increase at low temperature following the increase in mobility due to reduced lattice scattering^19,49^. As shown in Fig. 5c, the photoconductivity at 93 K for CsPbBr_3_ NPLs + PDI excited at 410 nm is one order of magnitude larger than of the NPLs alone and the lifetime also increases (Supplementary Fig. 15). In addition, in Fig. 5d it is shown that the maximum photoconductivity gradually increases upon lowering the temperature with the presence of the PDI molecules, while for the NPLs alone the photoconductivity is constant and up to an order of magnitude lower. These measurements unequivocally prove that the charge separation is efficient and mobile-free charge carriers (free holes) are formed when the PDI molecules are attached to the NPLs. Compared to other hybrid perovskites^19,50^, the maximum photoconductivity obtained ~0.07 cm^2^/Vs at 295 K and 0.2 cm^2^/Vs at 93 K (Supplementary Fig. 16) are 1–2 orders of magnitude lower. Possible reasons for this lower photoconductivity include confinement effects because of the lateral size of the platelets or differences in morphology compared to the crystalline thin films. As has been shown previously, the size of the domains in which charge transport occurs affects the microwave mobility that is measured if the charge carriers encounter the edges of these domains during the microwave oscillation cycle^19,51^. Nevertheless, the extremely long lifetime up to tens of microseconds (Supplementary Fig. 17), indicates the viability of this concept for application in opto-electronic devices. Long carrier lifetimes result in long diffusion lengths, which are essential for efficient charge collection by electrodes in solar cells. The latter requires that also the electrons trapped in the organic part of the materials can be transported over long ranges. The next step would therefore be the incorporation of conjugated molecules in solid-state 2D perovskites where such closely packed organic acceptors support electron transport.

## Discussion

In this work, we have demonstrated how improved functionality, in this case efficient charge carrier separation and long-range free carrier conduction, can be achieved in 2D perovskite NPLs by smart synthetic design attaching strong electron acceptors: amine-functionalized PDI molecules. By the unique combination of several ultrafast spectroscopy techniques, such as TA, picosecond PL spectroscopy and microwave conductivity measurements, we have shown that photoexcitation of either the NPLs or the PDIs leads to charge separation, and we have extracted the charge-transfer rates for electrons and holes (*k*_ET_ = 0.079 ps^−1^ and *k*_HT_ = 0.65 ps^−1^). Using microwave photoconductivity measurements, we show that the positive charges that are located in the perovskite NPLs can move around freely and have a long lifetime (tens of microseconds) that is sufficient for applications where charge extraction is required. To the best of our knowledge, this is the first time that improved charge separation has been directly shown in 2D perovskites by incorporation of a functional organic group in the same material (attached to the inorganic octahedral layer and not as heterojunction hampered by morphological issues). This opens up a new synergistic route to tune materials for specific applications by combining the inorganic perovskite layers with functional organic chromophores. Importantly, this concept is not limited to improved charge separation but one can also envision the use of functional organic molecules with chiral properties for circularly polarized light detection or advance processes such as singlet fission or up-conversion for enhanced light absorption.

## Methods

### Synthesis of CsPbBr_3_ NPLs

The colloidal CsPbBr_3_ NPLs with varying thickness were synthesized according to a previously reported protocol by Bohn et al.^26^ The ratio of the Cs-oleate and PbBr_2_ precursor, and the amount of antisolvent used, determines the thickness of the NPLs. To synthesize CsPbBr_3_ NPLs with 4 monolayers: 150 μL Cs-oleate precursor (prepared by dissolving 0.1 mmol Cs_2_CO_3_ in 10 mL oleic acid at 100 °C) was added to 1.2 mL PbBr_2_ precursor (prepared by dissolving 0.1 mmol PbBr_2_ in 10 mL toluene in the presence of 100 μL oleic acid and oleylamine at 100 °C) under continuous stirring. After ~5 s, 2 mL acetone was added, which initiated the crystallization of the NPLs. After 1 min, the solution was taken from the stirring plate, and the NPLs were centrifuged at 3500 rpm for 5 min in order to separate the crystalline NPLs from unreacted precursor, and the precipitate was re dispersed in 2 mL hexane. All syntheses were conducted under ambient conditions.

### Synthesis of *N-*(2,6-diisopropylphenyl)-*Nʹ*-(Boc-3-aminopropyl)-1,6,7,12-tetrachloroperylene bisimide (2)

A mixture of *N*-(2,6-diisopropylphenyl)-1,6,7,12-tetrachloroperylene monoimide monoanhydride **1** (0.50 g, 0.73 mmol, 1 eq.) and *N*-Boc-1,3-propanediamine (0.25 g, 1.45 mmol, 2 eq.) was taken in a round-bottom flask (50 mL) equipped with a water condenser. To this mixture, toluene (14 mL) was added. The combined mixture was refluxed for 18 h under argon atmosphere and then cooled to room temperature. Toluene was evaporated under vacuum and the solid residue was washed with water and methanol. Subsequently, the solid residue was dried and chromatographed on silica, with CH_2_Cl_2_ to afford the desired product (0.52 g, 85%)^34^. ^1^H NMR (400 MHz, CDCl_3_): *δ* = 8.74 (s, 2H), 8.71 (s, 2H), 7.51 (t, *J* = 8.1 Hz, 1H), 7.35 (d, *J* = 8.1 Hz, 2H), 5.10 (s, 1H), 4.31 (t, *J* = 6.4 Hz, 2H), 3.19 (m, 2H), 2.77‒2.68 (m, 2H), 2.00‒1.92 (m, 2H), 1.44 (s, 9H), 1.17 (t, *J* = 6.0 Hz, 12H). ^13^C NMR (100 MHz, CDCl_3_): *δ* = 162.485, 162.294, 155.926, 145.551, 135.584, 135.468, 133.378, 133.116, 131.616, 131.494, 129.988, 129.846, 128.849, 128.738, 124.232, 123.864, 123.339, 123.159, 123.058, 38.212, 37.568, 29.268, 28.514, 28.486, 28.423, 23.994. For synthesis path and nuclear magnetic resonance (NMR) spectrum, see Supplementary Figs. 3 and 4.

### Synthesis of *N-*(2,6-diisopropylphenyl)-*Nʹ*-(3-aminopropyl)-1,6,7,12-tetrachloroperylene bisimide (3)

Compound **2** (0.50 g, 0.59 mmol) was dissolved in DCM (10 mL)^34^. Trifluoroacetic acid (3 mL) was added to this solution. The combined reaction mixture was stirred for 1 h at room temperature. The progress of the reaction was thoroughly followed by TLC analysis of removed aliquots (10:1 DCM–EtOH). After complete consumption of the starting material, more DCM (100 mL) was added. The resultant solution was washed first with aqueous K_2_CO_3_ and then with water. The organic phase was collected and concentrated. The crude product was then chromatographed on silica with 10:1 DCM–EtOH mixture to yield the pure product (0.41 g, 93%). ^1^H NMR (400 MHz, CDCl_3_): *δ* = 8.74 (s, 2H), 8.71 (s, 2H), 7.52 (t, *J* = 8.1 Hz, 1H), 7.36 (d, *J* = 8.1 Hz, 2H), 4.34 (t, *J* = 6.8 Hz, 2H), 2.84 (s, 2H), 2.78‒2.68 (m, 2H), 1.98‒1.91 (m, 2H), 1.18 (t, *J* = 5.6 Hz, 12H). ^13^C NMR (100 MHz, CDCl_3_): *δ* = 162.378, 162.298, 145.551, 135.538, 135.468, 135.417, 133.374, 133.056, 131.618, 131.497, 129.986, 129.846, 128.877, 128.663, 124.230, 123.866, 123.329, 123.160, 123.130, 39.224, 38.329, 31.561, 29.266, 23.997. For synthesis path and nuclear magnetic resonance (NMR) spectrum, see Supplementary Figs. 3 and 5.

### Synthesis of CsPbBr_3_ nanoplatelets–perylene diimide hybrids

The PDI chromophores were attached to the CsPbBr_3_ NPLs by mixing the CsPbBr_3_ NPLs with PDI in DCM stock solution under vigorous stirring and mild heating (50 °C).

### Transmission electron microscopy

Transmission electron microscopy (TEM) images were acquired using a JEOL JEM-1400 plus TEM microscope operating at 120 kV. Samples for TEM imaging were prepared by drop-casting a dilute solution of NPLs onto a carbon-coated copper (400-mesh) TEM grid.

### Optical spectroscopy

Samples for optical measurements in solution were prepared by diluting the stock solution of washed CsPbBr_3_ NPLs with hexane. Absorption spectra were measured on a double-beam PerkinElmer Lambda 1050 UV/vis spectrometer. For fast-spectroscopy measurements, solutions with an optical density 0.1 at 400 nm of CsPbBr_3_ NPLs and ~0.14 at 510 nm of CsPbBr_3_ NPLs + PDI hybrid were prepared (Supplementary Fig. 2). PL spectra were recorded on an Edinburgh Instruments FLS980 Spectro-fluorimeter equipped with a 450 W xenon lamp as excitation source and double grating monochromators.

### Picosecond photoluminescence spectroscopy

The time-resolved fluorescence measurements are performed using Hamamatsu C5680 streak camera setup. A Chameleon Ultra II (Ti:Saph) oscillator (80 MHz) producing 140 fs pulses combined with a harmonic generator were used to excite the samples. Excitation wavelengths of 400 and 480 nm were used when exciting the NPLs and PDI, respectively. The overall time resolution of the setup is <10 ps.

### Femtosecond TA spectroscopy

Pump-probe femtosecond TA measurements were performed using a tunable laser system comprising a Yb:KGW laser source (1028 nm) operating at 5 KHz (2.5 KHz repetition rate) with a pulse duration of 180 fs (PHAROS-SP-06-200, Light Conversion) and an optical parametric amplifier (ORPHEUS-PO15F5HNP1, light conversion). Probe light was generated by continuum generation, focusing a small fraction of the fundamental laser light in a CaF_2_ crystal. The 2D data were acquired with a transient absorption spectrometer (HELIOS, Ultrafast Systems). The samples were placed in a 2 mm-path-length quartz cuvette and excited at 400 and 510 nm with pump fluences of ~3.5 × 10^13^ ph/(cm^2^ pulse) and (~1.9 × 10^13^ ph/(cm^2^ pulse)), respectively, and 200 μm probe spot size in quasi parallel pump-probe geometry.

### Time-resolved microwave photoconductivity measurements

Samples for time-resolved microwave conductivity (TRMC) measurements were prepared by drop-casting on quartz and placed in a sealed resonant cavity inside a helium-filled glovebox. The TRMC technique measures the change in microwave (8–9 GHz) power after pulsed excitation (repetition rate 10 Hz) at 400 and 510 nm in a temperature range of 295–93 K. The time resolution is limited by the width of the laser pulse (3.5 ns fwhm) and the response time of the system (18 ns). The slow repetition rate of the laser ensures full relaxation of all photoinduced charges to the ground state before the next laser pulse hits the sample. The product of the mobility $\left( {{\sum} {\mu = \mu _{\mathrm{e}} + \mu _{\mathrm{h}}} } \right)$ and dissociation yield of excitons (*x*) is calculated from the maximum photoconductivity $(\Delta G_{{\mathrm{max}}})$ according to Equation 1.

$$x{\sum} {\mu = \frac{{\Delta G_{{\mathrm{max}}}}}{{I_{\mathrm{o}}\beta eF_{\mathrm{A}}}}}.$$

## Supplementary information

Supplementary Information

## Data Availability

The Supplementary data of this study is available within the article and its Supplementary Information. The data that support the findings of this study are available from the corresponding author upon reasonable request.
