# Supplementary material for: Overcoming the exciton binding energy in two-dimensional perovskite nanoplatelets by attachment of conjugated organic chromophores
Source: Nat Commun. 2020 Apr 20;11:1901. doi: 10.1038/s41467-020-15869-7 (PMC7171160; doi:10.1038/s41467-020-15869-7)
Supplement: Supplementary file 1 — Supplementary Information [file 41467_2020_15869_MOESM1_ESM.pdf]

Supplementary Information

Overcoming the exciton binding energy in perovskite nanoplatelets by attachment of strong organic electron acceptors

María C. Gélvez-Rueda,[1] Magnus B. Fridriksson,[1] Rajeev K. Dubey,[1,2] Wolter F. Jager,[1] Ward van der Stam[1,3] and Ferdinand C. Grozema[1,*]

[1]Department of Chemical Engineering, Delft University of Technology, Van der Maasweg 9, 2629 HZ, Delft, The Netherlands.

[2]Present address: POLYMAT, Basque Center for Macromolecular Design and Engineering, University of the Basque Country UPV/EHU, Avenida de Tolosa 72, 20018, Donostia-San Sebastian, Spain

[3]Present address: Inorganic Chemistry and Catalysis, Debye Institute for Nanomaterials Science, Utrecht University, Universiteitsweg 99, 3584 CG, Utrecht, The Netherlands

Corresponding Author

*Ferdinand C. Grozema

E-mail: f.c.grozema@tudelft.nl

Supplementary Figures

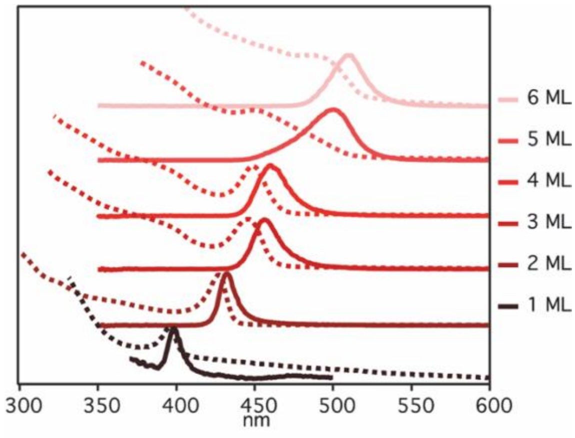

Supplementary Figure 1. Optical properties of $CsPbBr_3$ nanoplatelets as a function of thickness. Absorption (dashed lines) and photoluminescence (full lines) spectra of $CsPbBr_3$ nanoplatelets with varying thickness, ranging from 1 monolayer (1 ML) to 6 monolayers (6 ML).

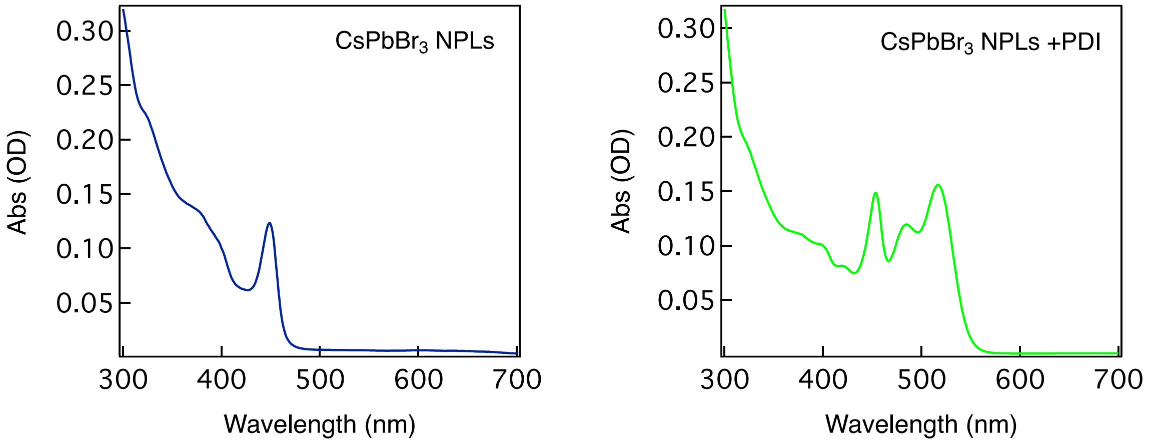

Supplementary Figure 2. Optical fraction of absorbed light. Optical fraction of absorbed light for $CsPbBr_3$ nanoplatelets (NPLs) (4 monolayers) and $CsPbBr_3$ NPLs + perylene diimide (PDI) hybrid.

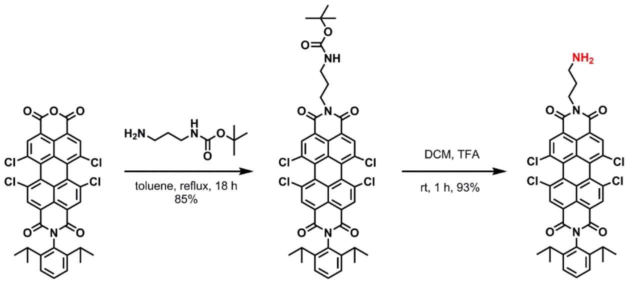

1 2 3

Supplementary Figure 3. Synthesis scheme of perylene diimide chromophores. Synthesis scheme of N-(2,6-diisopropylphenyl)-N'-(3-aminopropyl)-1,6,7,12-tetrachloroperylene bisimide (3).

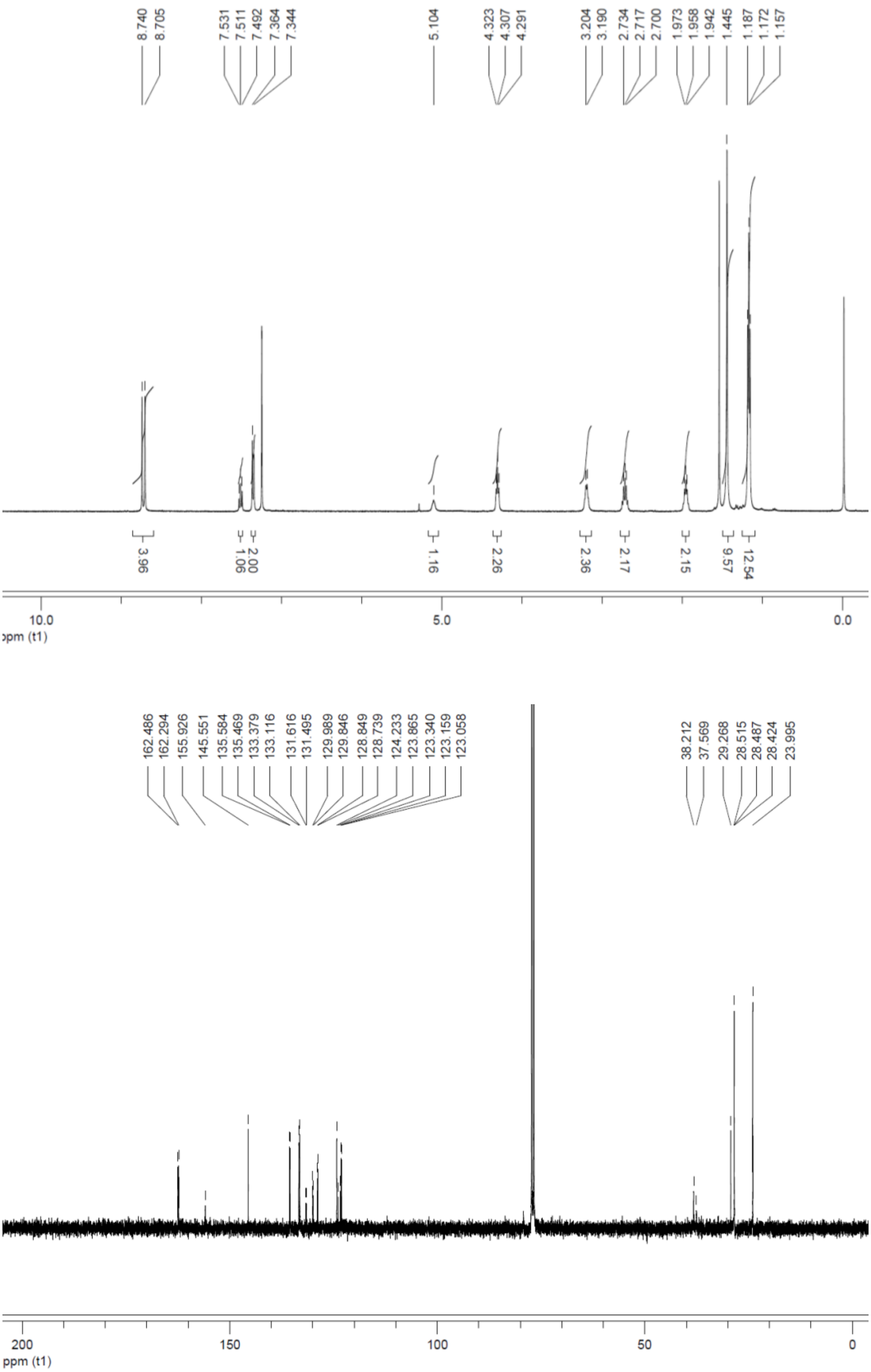

Supplementary Figure 4. ^{1}H and ^{13}C NMR spectra. ^{1}H and ^{13}C NMR spectra of compound **2** in $CDCl_3$.

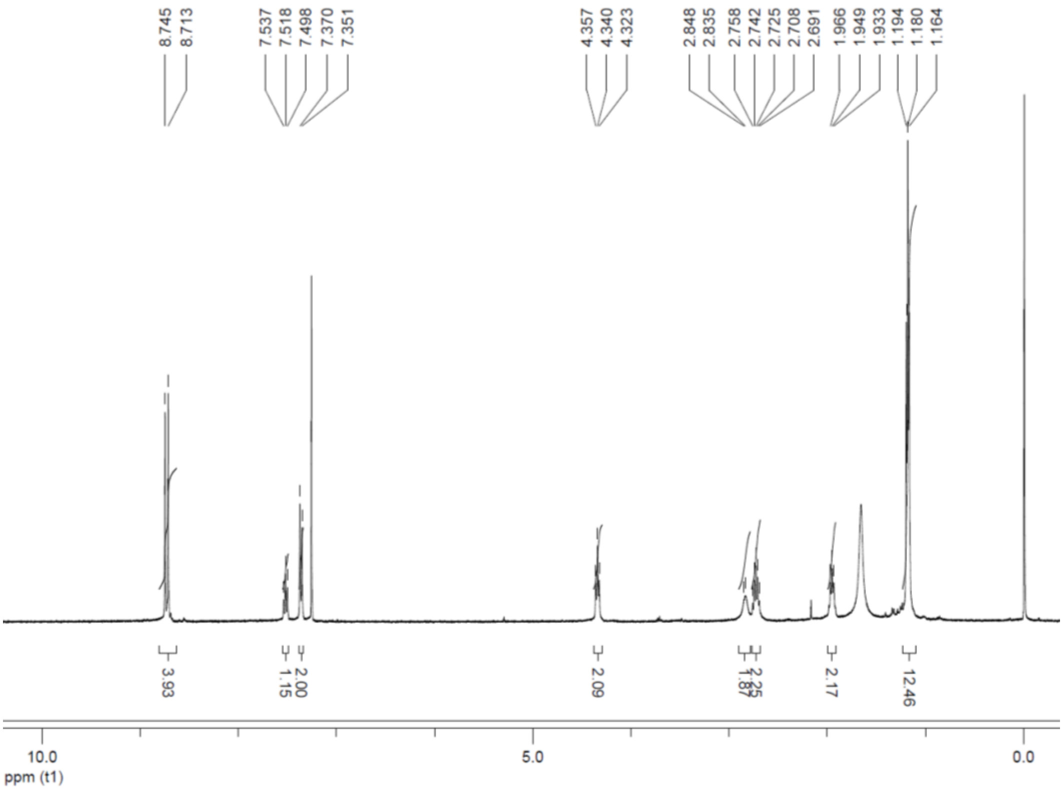

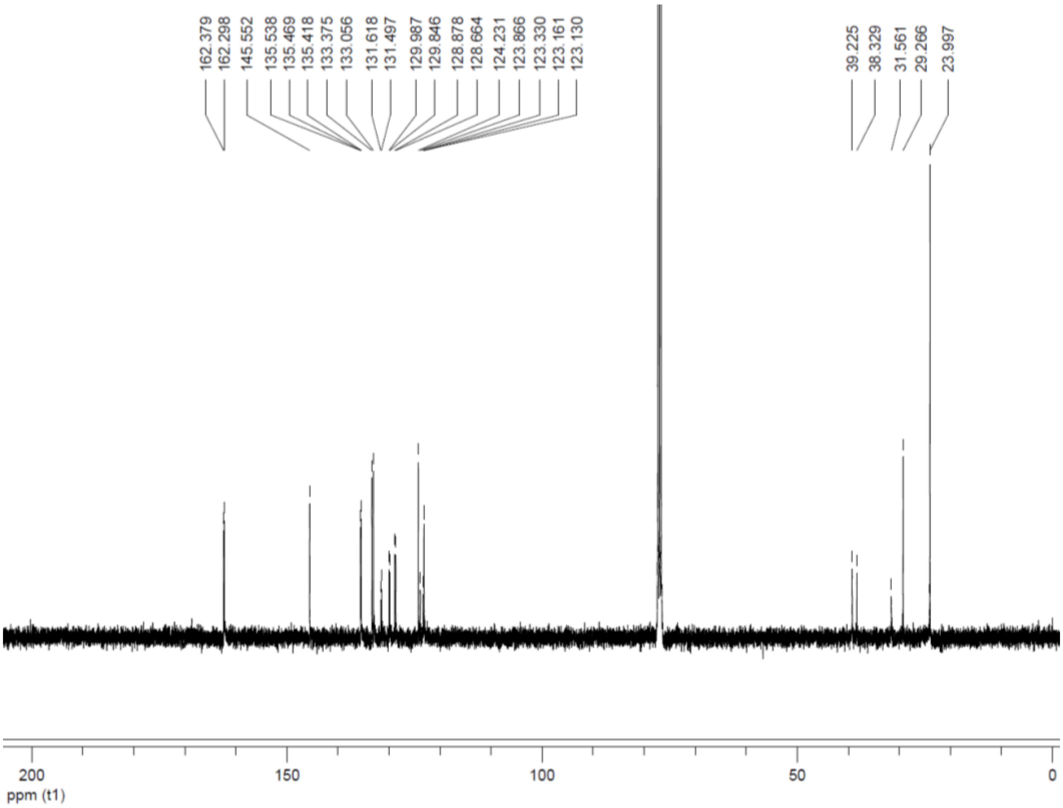

Supplementary Figure 5. ^{1}H and ^{13}C NMR spectra. ^{1}H and ^{13}C NMR spectra of compound **3** in $CDCl_3$.

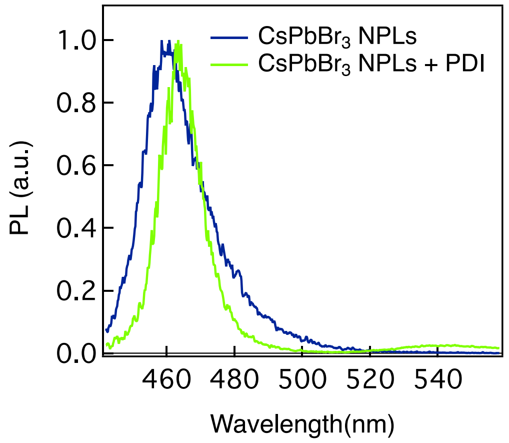

Supplementary Figure 6. Photoluminescence emission slice upon excitation at 400 nm. Photoluminescence emission slices of $CsPbBr_3$ nanoplatelets (NPLs) and $CsPbBr_3$ NPLs + PDI hybrid excited at 400 nm at 60 ps (spectral range 450 nm to 550 nm).

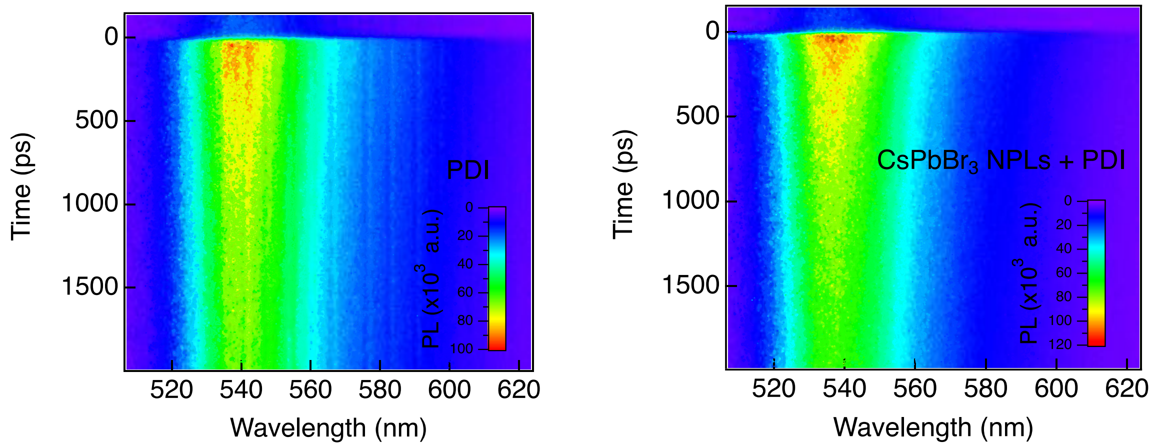

Supplementary Figure 7. Photoluminescence emission spectra on photoexcitation at 400 nm. Photoluminescence emission spectrum of perylene diimide (PDI) and $CsPbBr_3$ nanoplatelets (NPLs) + PDI hybrid in time upon exciting at 400 nm (spectral range 500 nm to 625 nm).

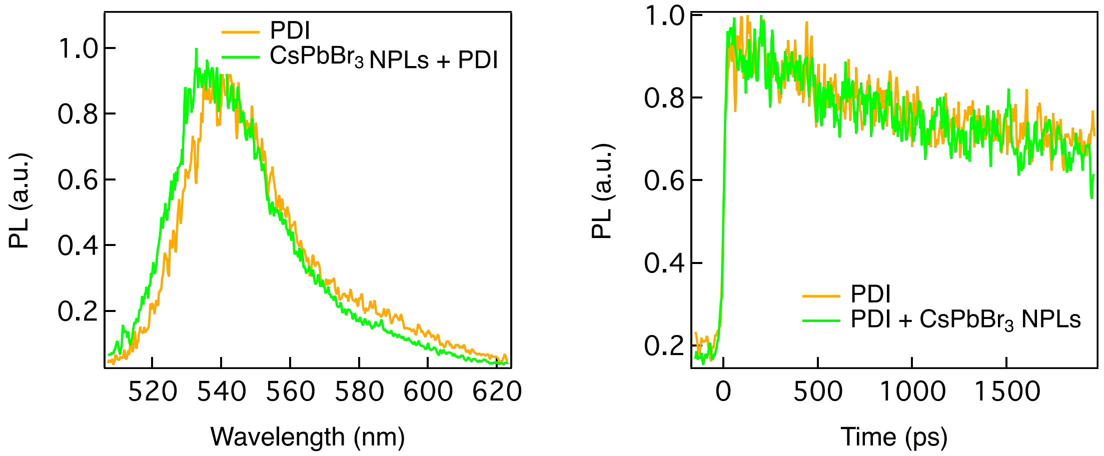

Supplementary Figure 8. Photoluminescence emission and temporal decay slices on photoexcitation at 400 nm. Photoluminescence emission slices of perylene diimide (PDI) and $CsPbBr_3$ nanoplatelets (NPLs) + PDI hybrid upon exciting at 400 nm and at a time of 60 ps (spectral range 500 nm to 625 nm). Comparison of the temporal decay of PDI and $CsPbBr_3$ NPLs + PDI hybrid at 540 nm (time range up to 1800 ps).

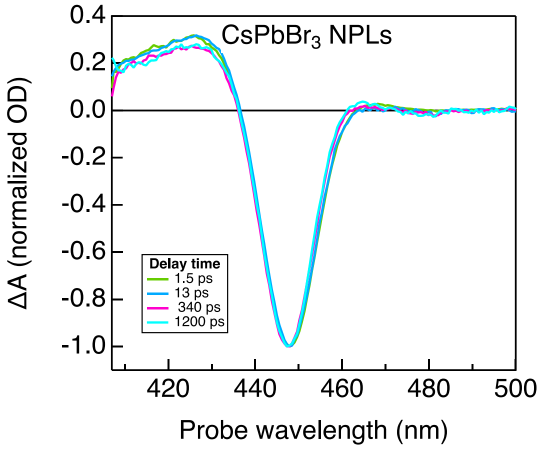

Supplementary Figure 9. $CsPbBr_3$ nanoplatelets normalized transient absorption spectrum on excitation at 400 nm.

Normalized transient absorption (TA) spectrum of $CsPbBr_3$ nanoplatelets (NPLs) in time upon exciting at 400 nm.

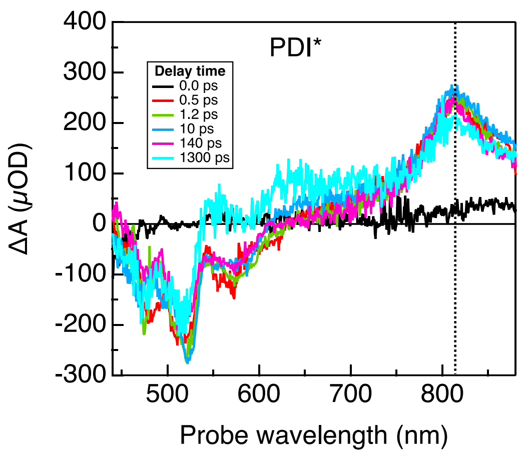

Supplementary Figure 10. Perylene diimide chromophores transient absorption spectrum on photoexcitation at 400 nm.

Transient absorption (TA) spectrum of free perylene diimide (PDI) chromophores in solution upon exciting at 400 nm.

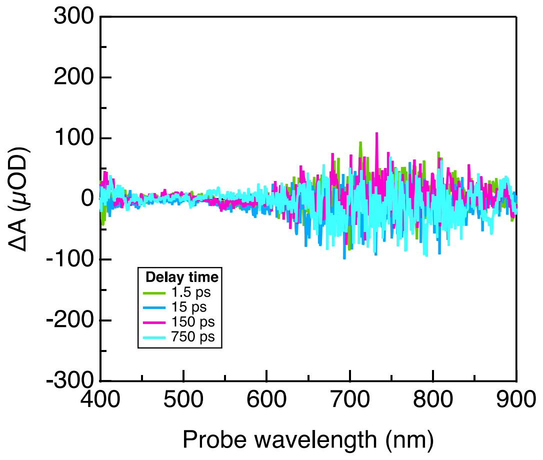

Supplementary Figure 11. $CsPbBr_3$ nanoplatelets transient absorption spectrum on photoexcitation at 510 nm. Transient absorption (TA) Spectrum of free CsPbBr3 nanoplatelets (NPLs) in hexane upon exciting at 510 nm.

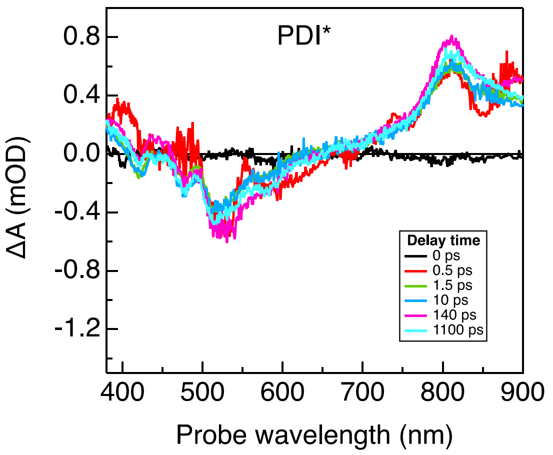

Supplementary Figure 12. Perylene diimide chromophores transient absorption spectrum on photoexcitation at 510 nm.

Transient absorption (TA) spectrum of free perylene diimide (PDI) molecules in hexane upon exciting at 510 nm.

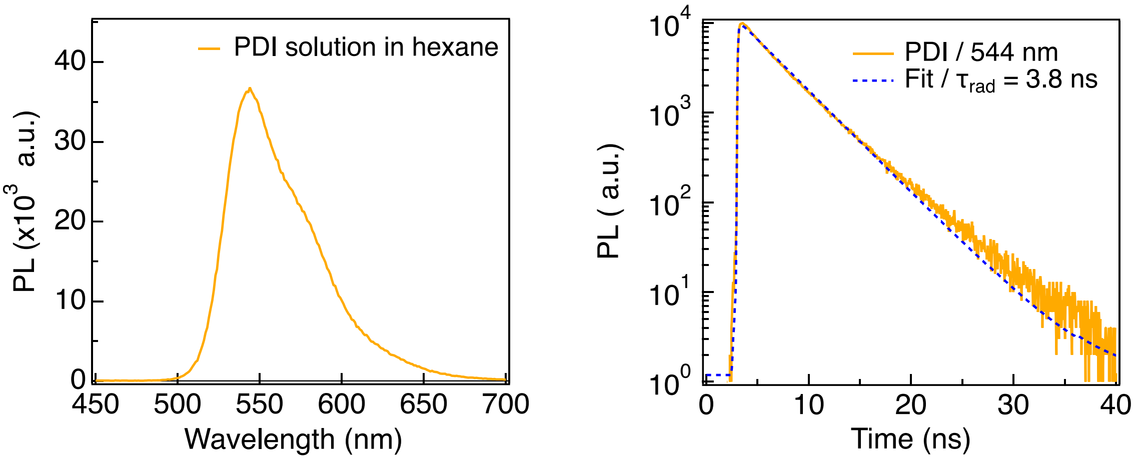

Supplementary Figure 13. Perylene diimide photoluminescence emission spectrum and temporal decay. Photoluminescence emission spectrum (spectral range from 450 to 700 nm) and temporal decay lifetime (time range up to 40 ns) of perylene diimide (PDI) chromophores in hexane upon exciting at 405 nm.

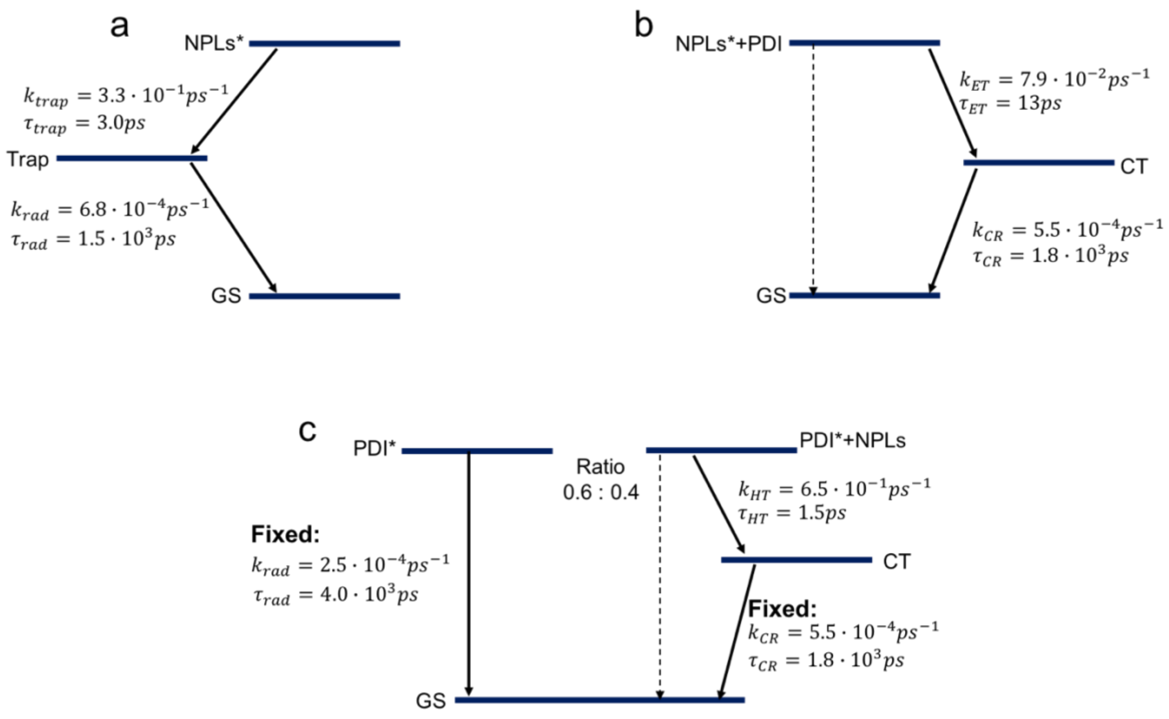

Supplementary Figure 14. Global and target analysis models. Global and target models used along with the fitted rates. **a,** $CsPbBr_3$ nanoplatelets (NPLs) upon exciting at 400 nm. **b**, electron transfer from $CsPbBr_3$ NPLs* to perylene diimide (PDI) upon exciting at 400 nm. **c,** hole transfer from PDI* to $CsPbBr_3$ NPLs upon exciting at 510 nm.

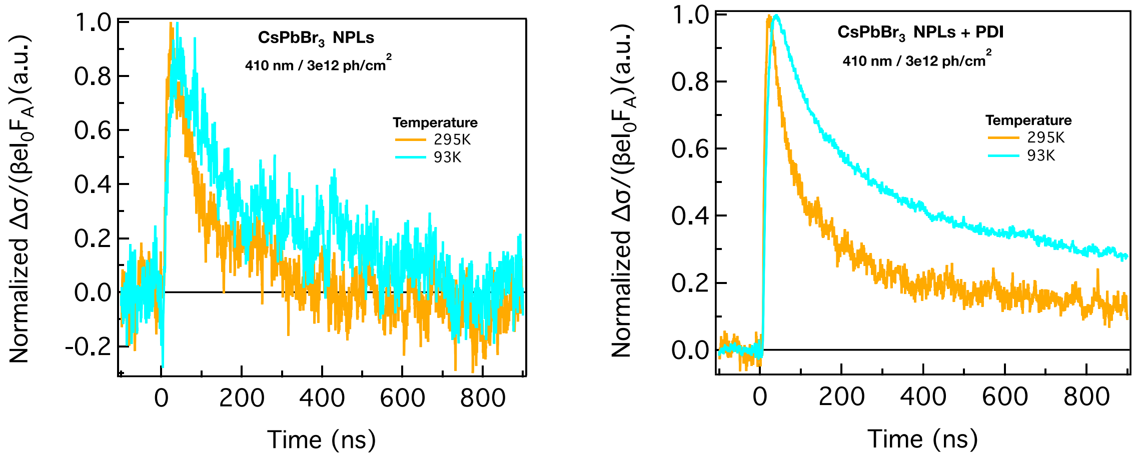

Supplementary Figure 15. Normalized photoconductivity in time. Normalized photoconductivity of $CsPbBr_3$ nanoplatelets (NPLs) and CsPbBr3 NPLs + perylene diimide (PDI) hybrid at 295K and 93K.

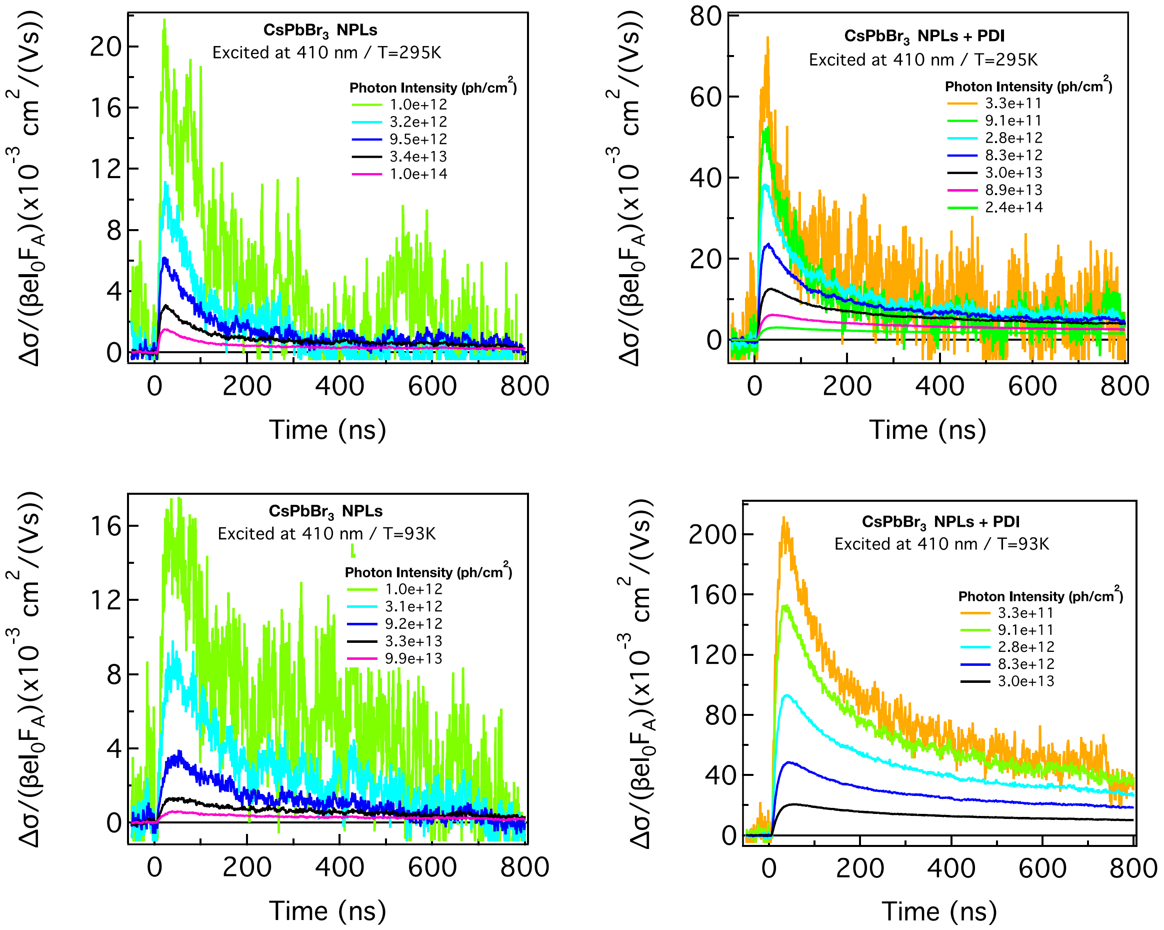

Supplementary Figure 16. Photoconductivity in time as a function of photon intensity. Photoconductivity in time as a function of photon intensity CsPbBr3 nanoplatelets (NPLs and CsPbBr3 NPLs + perylene diimide (PDI) at 295K and 93K.

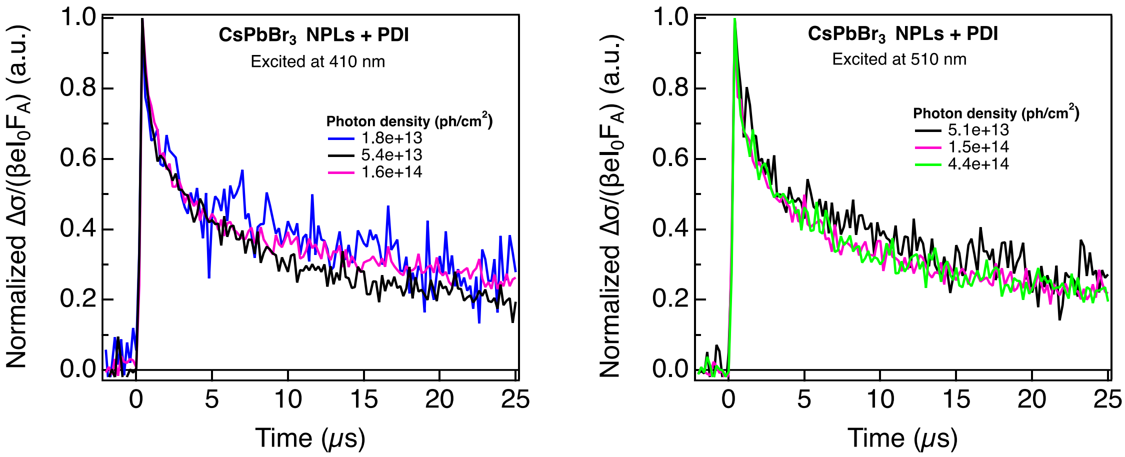

Supplementary Figure 17. Normalized photoconductivity in microsecond time scale. Normalized photoconductivity of $CsPbBr_3$ nanoplatelets (NPLs) + perylene diimide (PDI) hybrid at 295K and upon excitation at 410 nm and 510 nm (microsecond time scale).

Supplementary Notes

Supplementary Note 1. Optical Properties of $CsPbBr_3$ Nanoplatelets (n=1 to n=6)

The monodispersity of the 4 monolayer (ML) $CsPbBr_3$ nanoplatelets (NPLs) used is corroborated by transient electron microscopy (TEM) analysis (inset Figure 1b) and by the steady-state absorption spectrum (Figure 1b) which displays very narrow band edge absorption (full-width-half-maximum ~100 meV). The absorption spectrum shows additional maxima at higher energy. This is typically observed for strongly confined nanomaterials, since the linear combination of the atomic orbitals results in a quasi-continuum in the conduction band, and discrete energy levels near the band edges, which also explains the narrow band edge absorption. If substantial amounts of thinner nanoplatelets would be present in the ensemble, narrow absorption features would be superposed on the absorption spectrum of the n=4 nanoplatelets at wavelengths of 398 nm, 430 nm, and 450 nm, for n=1, 2 and 3 nanoplatelets. The absorption shoulders in the spectrum of the n=4 nanoplatelets are located at 390 nm and 410 nm, already suggesting that they do not correspond to n=1 and 2 subpopulations. In addition, if these thin NPLs would have been present, photoexcitation at 400 nm would results in distinct bleach features for these thinner NPLs in the TA, especially on short time scales before energy transfer can occur.

Supplementary Note 2. Estimation of Number of Perylene Diimide Chromophores on the $CsPbBr_3$ Nanoplatelets Surface

We have estimated the number of perylene diimide (PDI) chromophores coordinated to the $CsPbBr_3$ nanoplatelets (NPLs). However, we stress that this is a rough estimation made from the size of the NPLs determined by transient electron microscopy (TEM), intrinsic absorption coefficient (μ_i) of $CsPbBr_3$ nanocrystals[1] (same size than the NPLs and same solvent), concentration and volume of PDI solution added to the NPLs solution, and the ratio from attached PDI molecules and free PDI molecules in solution obtained from the fit of the TA data at 510 nm by global target analysis.

First, it is possible to determine the volume of nanoplatelets (V_{NPLs}) in the solution measured from the intrinsic absorption coefficient according to Supplementary Equation 1. However, the intrinsic absorption coefficient of $CsPbBr_3$ NPLs is not known. We assumed that this coefficient is similar to the intrinsic absorption coefficient of $CsPbBr_3$ nanocrystals with a similar lateral size (4-5 nm) and determined in the same solvent used in our experiments (Hexane).[1] In fact, Maes et al.[1] determined that the intrinsic absorption coefficient is independent of nanocrystal size and is approximately 77000 cm^{-1} at 400 nm in Hexane.

$$\mu_i = \frac{\ln 10 * A}{f * L} \qquad 1$$

Where, A is the absorbance (optical density), f the volume fraction (V_{NPLs} / $V_{Dispersion}$) and L the optical path length. From the solution used for the steady state absorption shown in Supplementary Fig. 2, A=0.1 at 400 nm, $V_{Dispersion}$=3.3 cm^3, and the optical path length of the cuvette is 1 cm.

Then, from Supplementary Equation 1 the volume of nanoplatelets (V_{NPLs}) is approximately $1.32x10^{-6}$ cm^3.

The volume of each nanoplatelet can be determined from the size dimensions measured by TEM (NPLs dimensions= 5 x 10 x 1.5 nm) resulting in V_{NPL}= 7.5 $x10^{-20}$ cm^3. In consequence, the number of nanoplatelets is ~$1.312x10^{14}$ NPLs. Leading to a concentration of $4x10^{13}$ NPLs/cm^3.

Second, the PDI solution added was 0.1 cm^3 at a concentration of $5x10^{-4}$ M. This results in a total number of PDI molecules added of $3.01x10^{16}$ PDIs. From the fitting of the TA data exciting at 510 nm it was determined that the percentage of PDI molecules attached to the platelets is approximately 40% ($1.204x10^{16}$ PDIs). Using the number of nanoplatelets determined above, it is determined that the number of attached PDI molecules per nanoplatelet is ~91. This value is reasonable as is highly likely that on the surface of the

nanoplatelets there are also oleic acid anionic ligand (on Cs^+ states), while the PDI molecules would act as cationic ligands on (on PbBr states).

Supplementary Note 3. Glotaran Global Target Analysis

The global and target analysis is performed with the Glotaran software.[2] We have modelled the radiative recombination of both the $CsPbBr_3$ nanoplatelets (NPLs) and the perylene diimide (PDI) chromophores in solution; and also the electron and hole transfer between the $CsPbBr_3$ NPLs + PDI hybrid. Here we will describe each model along with the assumptions used and list the results. The models and the fitted rates are summarized in Supplementary Fig. 14.

Perylene diimide excited state kinetics

The radiative recombination of the perylene diimide (PDI) chromophores was modelled with a simple single rate constant model. The decay of the PDI excited state (~4ns)[3] is considerably longer than the time-window of the TA experiment, making it impossible to derive an accurate estimate of the decay time. Therefore, we measured the fluorescence lifetime of PDI molecules in solution on longer timescales by time-correlated single photon counting (TCSPC) using a Lifespec fluorescence lifetime spectrometer (Edinburgh Instruments). The resulting fluorescence lifetime (3.8 ns) was used as a fixed decay time for PDI* in the global analysis. Supplementary Figure 13.

$CsPbBr_3$ nanoplatelets excited state kinetics

If a similar single rate constant model is used for the $CsPbBr_3$ nanoplatelets (NPLs) the model fails to fit the early timescales. Therefore, a sequential two rate constant model is used where the short lifetime of 3 ps is assigned to shallow trapping and the longer lifetime of 1.5 ns as the radiative recombination rate.

$CsPbBr_3$ nanoplatelets + Perylene diimide hybrid exciting the nanoplatelets at 400 nm

In order to keep the model for the electron transfer from the nanoplatelets (NPLs) to the Perylene diimide (PDI) chromophores as simple as possible we again model it with a sequential two rate constant model. This first of all assumes that every NPL is connected to a PDI which is reasonable as the concentration of PDI molecules is much higher than the concentration of NPLs. The second thing to note about this model is that the fast rate constant in this model will not only be influenced by the electron transfer rate but also, to a small extent, the fast trapping in the NPLs. The second rate constant will then describe the charge recombination from the charge transfer state. We obtain an electron transfer rate of 12.6 ps and charge recombination of roughly 1.8 ns.

$CsPbBr_3$ nanoplatelets + Perylene diimide hybrid exciting the chromophores at 510 nm

When the hole transfer from the Perylene diimide (PDI) to the $CsPbBr_3$ nanoplatelets (NPLs) is modelled a more complex model has to be used. Since the concentration of PDI is much higher than that of the NPLs there will be two different populations of PDI; (1) PDI connected

to a NPL which will undergo hole transfer and (2) free PDI in solution that will decay radiatively as described above. The ratio between these populations is unknown and has to be modelled along with the hole transfer rate. The radiative recombination rate is assumed to be 3.8 ns as previously mentioned and the charge recombination rate is taken to be the same as it was for recombination of CT states formed by electron transfer. The ratio between the two PDI populations that we obtain is 6:4 and the hole transfer rate is 1.5 ps.

Supplementary Note 4. Efficiency of electron and hole transfer

We estimated the efficiency of charge transfer from the TA measurements at 400 nm. At 400 nm, only the $CsPbBr_3$ nanoplatelets (NPLs) are excited and any TA features from the PDI are a result of electron transfer to the Perylene diimide (PDI) chromophores. We estimated the efficiency of electron transfer by determining the concentration of PDI molecules in the excited state ($[PDI^*]$) from the magnitude of the bleach or Absorbance (A) of the PDI molecules at ~520 nm and comparing it to the absorbed photons at 400 nm after pump excitation. We assume that the $[PDI^*]$ is equal to the concentration of PDI anions ($[PDI^-]$) as the PDI molecules are not excited at 400 nm. In addition, the extinction coefficient of PDI anion with chlorines in the bay area is not known. Using The Beer-Lambert Law:

$$A_{520nm} = \varepsilon_{520\,nm}\,[PDI^*]\;L$$

Where, A is the TA absorbance optical density, ε is the extinction coefficient of the PDI in the excited state and L the optical path length in the TA experiments. From the TA results at 400 nm, the absorbance of the PDI excited state at 520 nm is ~ 1.5×10^{-4} OD and the optical path length is 0.2 cm. The extinction coefficient for the PDI compound with chlorine in the bay has been previously determined to be ~50000 $cm^{-1}M^{-1}$ at 520 nm.[3] From (2), $[PDI^*]$ is approximately ~1.5×10^{-8} M.

As mentioned in the TA experimental condition, the absorbed photon intensity at 400 nm is ~3.5×10^{12} ph/cm^2. To determine the photon concentration in M (mol/L), this value is divided by the optical path length and Avogadro's number resulting in an initial concentration of absorbed photons of ~2.9×10^{-8} M. Comparing the concentration of PDI excited states to the initial concentration of photons, the efficiency of electron transfer into the PDI molecules is ~ 52%.

From the TA experiments at 510 nm is not possible to determine directly the efficiency of hole transfer as the extinction coefficient of the PDI anion at ~760 nm is not known. It is possible to estimate an efficiency of hole transfer from the bleach of the PDI excited state at 520 nm, using the TA fit from the global analysis (40 % PDI molecules are attached). So $[PDI^*]$*0.4 =

~1.5x10^{-8} M, while the absorbed photons at 510 nm are ~2.24x10^{-8} M. Therefore, the efficiency of hole transfer into the NPLs is ~ 54%. This efficiency is rather low considering that the time constant of hole transfer from the TA global analysis is one order of magnitude faster than of electron transfer.

Supplementary Note 5. Driving force for electron and hole transfer (ΔG)

Although the reduction potential of Perylene diimide (PDI) molecules is known (~2.5 -2.7), this is not the case for the oxidation potential of $CsPbBr_3$ nanoplatelets (NPLs). Estimations of the conduction and valence band to vacuum of $CsPbBr_3$ has been made in literature from the band positions of 3D $MAPbBr_3$ by assuming that the bands are equally formed by Pb and Br orbitals shifted by 2D confinement and image-charge effects. This estimation is rather rough and attempts to calculate the position of the charge separated state of barrier energy result in inadequate energy states (CS state above the excited states of both NPLs and PDI). The reason for this mismatch is that the charge stabilization energy from the solvent is not included, neither on the PDI molecules or on the $CsPbBr_3$ NPLs. This stabilization energy can be calculated on the PDI molecules but is highly sensible to the radio taken for the point charge model. This estimation is not so simple on the $CsPbBr_3$ NPLs in addition to the holes being delocalized over the NPLs size.

Our experimental results show that the relative ordering of the energy levels in Figure 1c is correct for the energetic environment in our system. The only absolute statement we can make is that the driving force for electron transfer is at least 350 meV.

Supplementary References

1. Maes, J. *et al.* Light Absorption Coefficient of CsPbBr3 Perovskite Nanocrystals. *J. Phys. Chem. Lett.* **9**, 3093–3097 (2018).

2. Snellenburg, J. J., Laptenok, S. P., Seger, R., Mullen, K. M. & van Stokkum, I. H. M. Glotaran : A Java-based graphical user interface for the R package TIMP. *J. Stat. Softw.* **49**, 1–2 (2012).

3. Dubey, R. K., Westerveld, N., Sudhölter, E. J. R., Grozema, F. C. & Jager, W. F. Novel derivatives of 1,6,7,12-tetrachloroperylene-3,4,9,10-tetracarboxylic acid: Synthesis, electrochemical and optical properties. *Org. Chem. Front.* **3**, 1481–1492 (2016).
